# A Software-Defined Networking Framework to Provide Dynamic QoS Management in IEEE 802.11 Networks

**DOI:** 10.3390/s18072247

**Published:** 2018-07-12

**Authors:** Pilar Manzanares-Lopez, Josemaria Malgosa-Sanahuja, Juan Pedro Muñoz-Gea

**Affiliations:** Department of Information and Communication Technologies, Universidad Politecnica de Cartagena, 30202 Cartagena, Spain; josem.malgosa@upct.es (J.M.-S.); juanp.gea@upct.es (J.P.M.-G.)

**Keywords:** wireless networks, SDN, QoS, mathematical modeling

## Abstract

In this paper, the concept of SDN (*Software Defined Networking*) is extended to be applied to wireless networks. Traditionally, in a wired SDN environment, the OpenFlow protocol is the communication protocol used to configure the flow table of forwarding elements (i.e., switches and Access Points). However, although in IEEE 802.11 networks there is no concept of forwarding, the SDN paradigm could also be applied to set up the wireless network dynamically, in order to improve the performance. In this case, not only the network elements, that is the Access Points, but also the mobile elements should configure their link and physical layers parameters following the guidelines of a centralized SDN controller. In particular, we propose a mechanism called DEDCA (*Dynamic Enhanced Distributed Channel Access*) to manage the channel access in wireless networks, and a framework that enables its implementation in 802.11-based wireless networks using SDN technology. The key aspect of this alternative solution is the control over the contention window size of the wireless terminals. Thus, an adequate response to dynamic and short-term Quality of Service (QoS) requirements can be offered to services running on these networks. DEDCA mechanism relies upon the use of a scalar parameter called *gain*. The mathematical model which has allowed us to obtain this parameter is presented and evaluated in this paper. Finally, the usefulness of the proposed solutions have been evaluated by means of their implementation in an example case.

## 1. Introduction

Wireless networks have experimented an exponential growth in the last few years. Currently, wireless is the most used technology by devices to access to the Internet or to communicate with one another or throughout an access point (AP). Wireless networks are also being used in other environments, which are very sensitive in terms of Quality of Service (QoS), such as Vehicular Ad-Hoc Networks (VANET) or Wireless Sensor Networks (WSN). In addition, it is expected that low-cost Single Board Computer (SBC) devices like Raspberry-pi or Odroid produce a further expansion of wireless networks, especially for real-time applications in IoT environments, which are also QoS aware. As a consequence, nowadays, the QoS in wireless networks is no longer an option but a requirement.

One of the methods to provide QoS in multiple access networks is by giving more channel access opportunities to nodes involved in QoS-aware sessions, using the Binary Exponential Backoff mechanism. In fact, this is the background idea of the IEEE 802.11e in its contention-based medium access function, called *Enhanced Distributed Channel Access* (EDCA) [1], which allows traffic differentiation for the stations in the network. EDCA operation is based on the idea of four Access Categories (AC): Voice, Video, Best Effort, and Background. Access Categories map directly from Ethernet-level class of service (CoS) priority levels, defined in IEEE 802.1p.

IEEE 802.11e provides long-term QoS in terms of application requirements. Different backoff values are defined for each access category, according by the physical layer supported by IEEE 802.11e. However, these values are fixed and cannot be dynamically modified to maintain the QoS requirements against network changes or to response to dynamic application needs.

In this regard, we can highlight one of the contributions of this work. We propose DEDCA (*Dynamic Enhanced Distributed Channel Access*) mechanism for 802.11 networks. This mechanism offers an efficient solution for the dynamic management of the QoS requirements of terminals. In our proposal, independently of the kind of traffic, the configuration of the backoff values is not fixed over the time, but they depend on the punctual QoS requirements of the applications.

Being able to respond to QoS requirements of applications in short-term intervals can be useful, especially in some scenarios such as VANET, WSN and IoT (Internet of Things). This new scenario of short-term QoS requirements could be solved by giving a light advantage to these particular stations assigning, only temporarily, more bandwidth to them at the expense of reducing the bandwidth of other nodes. This is the key idea of mechanism presented in this work.

To perform this new QoS aware access method, a mathematical model to orchestrate who can—and who cannot— temporarily use an advantageous backoff procedure would be very valuable. Unfortunately, the mathematical analysis proposed in the literature to model 802.11 networks (most of them based on Markov chains) are too complex to be used for this purpose. In this paper it is proposed an easy-to-use mathematical model, which allows the development of new QoS policies able to adapt to the network dynamics in real time. The mathematical model, another contribution of this work, relies only on the calculation of a scalar value called *gain*. This solution is computationally much simpler than most of the solutions proposed in the literature, and consequently can be useful even for low-performance devices like embedded systems. The mathematical model is in fact an extension of a preliminary study that was developed for PLC networks (*Power Line Communications*) [2]. Here, the model has been extended to enable that all the nodes can configure different levels of QoS independently, by the modification of its QoS related configuration parameters. As far as we know, there is not any other mathematical model in the literature with these characteristics.

In addition, we propose a SDN (*Software Defined Networking*)-based framework that takes advantage of the centralized management of the network to give a response to dynamic QoS requirements in 802.11 networks by applying the DEDCA mechanism. Traditionally, SDN is in charge of configuring the wired networks by dynamically changing the flow table of a forwarding element (i.e., *router* or *switch*) [3]. On the other hand, when SDN is applied to the wireless networks, it is mainly used to configure a set of APs for efficient resource allocation, interference mitigation, network slicing, congestion control, and load balancing among other things [4]. However, in this SDN framework, not only the Access Points but also the mobile elements will be able to configure their link and physical layers parameters following the guidelines of a centralized SDN controller. The SDN controller monitors the mobile devices and, depending the QoS requirements, the backoff values of some of them will be modified in real time, following the rules of the developed mathematical model.

The amount of real scenarios and applications which could benefit from the implementation of the proposed DEDCA mechanism is wide and varied. By way of example, we are going to consider a particular scenario that allows us to show the validity and usefulness of our proposal. In this case, the proposed SDN-based framework is used to manage a controlled surveillance system in an outdoor parking lot.

Our proposal has been evaluated by simulation using Estinet network simulator. The testbed consists of an SDN 802.11a Access Point and a set of 802.11a outdoor surveillance cameras, that are controlled by a SDN controller. The Wifi-Infrastructure module of Estinet 9.0 has offered us the possibility of modifying the IEEE 802.11a protocol configuration as required.

The rest of the paper is organized as follows: Section 2 presents the DEDCA (*Dynamic Enhanced Distributed Channel Access*) mechanism and describes the mathematical model it is based on. The SDN-based framework for IEEE 802.11 infrastructure-based networks that allows the application of the DEDCA mechanism is presented in Section 3. In Section 4 the mathematical model and the applicability of the proposed solutions in a specific scenario are evaluated by simulation. A review of related works are described in Section 5. Finally, Section 6 summarizes the main contributions of this work.

## 2. Contention Window Adaptation Mechanism

### 2.1. The Backoff Scheme in Shared Networks

The well-known wireless network IEEE 802.11 family, especially the IEEE-802.11e, provides a QoS compliant medium access control based on traditional CSMA/CA. A common aspect of any kind of CSMA/CA variant is the exponential backoff procedure. Actually, most of the QoS facilities in wireless networks are provided by means of fine-tuning -in one way or in another- the behavior of the exponential backoff scheme (see Related Work section for more information).

The traditional exponential backoff scheme consists of generating a random backoff interval to minimize the probability of collision with other contending stations. For every packet transmission, the backoff interval is obtained from the slot time and a value called backoff counter. The backoff counter is uniformly chosen in the range (0, CW), where CW is called *Contention Window*. CW value is related to the number of failed transmission attempts for a packet. Initially, CW is set equal to a value $CW_{min}$, called the minimum contention window. For each unsuccessful transmission, CW is doubled up to a maximum value $CW_{max}=2^{m}CW_{min}$. On the other hand, the backoff counter is decremented as long as the channel is sensed idle. If a transmission is detected on the channel, the counter is frozen, and reactivated when the channel is sensed idle again. When the counter reaches 0, the station transmits. A successful packet reception is marked by the transmission of a positive acknowledgment (ACK) by the destination station after another fixed period of time (*Short Interframe Space* (SIFS)). The failure in the reception of an ACK frame at the transmitter is assumed as a collision at the receiver. On the other hand, upon a successful transmission, the backoff algorithm reduces the contention window to $CW=CW_{min}$. The backoff procedure guarantees a fair play (i.e., the channel is equally shared among all station) and, at the same time, it tries to avoid collisions.

Using the exponential backoff mechanism is one of the methods of providing QoS in multiple access networks. Depending on the values of the backoff parameters, a mobile terminal is giving more channel access opportunities. This is the background idea of the IEEE 802.11e in its contention-based medium access function, called *Enhanced Distributed Channel Access* (EDCA) [1]. 802.11e defines different types of user data named Access Categories (AC): Voice, Video, Best Effort, and Background, and defines $CW_{min}$ and $CW_{max}$ values of contention window depending on the type of data. These values are fixed and cannot be dynamically changed to maintain the QoS requirements against network changes or to response to dynamic application requirements.

### 2.2. The Proposed Mechanism

In this work, we propose DEDCA (*Dynamic Enhanced Distributed Channel Access*) mechanism, an alternative solution to response to dynamic QoS requirements in 802.11 network based on the definition of Terminal Categories (TC). By default, all the terminals are classified as *normal stations*, using default backoff values. When a terminal requests a punctual and time-limited more demanding QoS requirement it becomes a *requesting station*. The *requesting station* will modify its $CW_{min}$ value to increment the channel access opportunity and, consequently to obtain more bandwidth, at the expense of other terminals that give some bandwidth up to the *requesting station*. These last terminals, that also modify their $CW_{min}$ value to decrement their channel access opportunities, are called *giving terminals*.

With the goal of determining how to modify the contention window size of the *giving* and *requesting stations*, a mathematical model has been developed. At this point, it is important to stress that we are not interested in finding a general purpose mathematical model of shared networks using the backoff mechanism. On the contrary, we are looking for a simple and easily programmable model that offers a tool to manage the QoS of these networks. Concretely, we will find a scalar called *gain* (*G*), which will show us how the bandwidth is spread out among the wireless terminals.

In this paper, we present a particular implementation of this proposed mechanism. As presented in Section 3, we propose to make use of the *Software Defined Networking* (SDN) technology to define a framework where DEDCA mechanism can be applied in order to give a response to dynamic QoS requirements in 802.11 network.

#### 2.2.1. Modeling the Gain

In backoff-based algorithms, the stations that are competing for the medium have a probability of winning the channel that depends on the backoff parameters. We call this probability P(win). As said before, DEDCA algorithm is based on the modification of the $CW_{min}$ value of some terminals to increase or decrease their probability of winning the channel.

Thus, we define *Gain* *G* as the multiplicative increase or decrease of this probability when a terminal modifies its $CW_{min}$ value with respect to a non-modified terminal. This scalar value *G* is obtained as the probability of winning the channel when the station modifies its $CW_{min}$ value, normalized to the case when $CW_{min}$ has the default value:

$$G=\frac{{P\left(win\right)|}_{CW_{min}=default\_CW_{min}\pm k}}{{P\left(win\right)|}_{CW_{min}=default\_CW_{min}}}$$

Firstly, we obtain the probability of winning the channel access of a reference station, that is, a non-modified station.

The $U_{i}$ variable is defined as the backoff counter that station *i* obtains for a particular channel access. For convenience, instead of using the interval $\left[0,CW_{min}\right]$ we will use the interval [1,W], where $W=CW_{min}+1$. Therefore, $U_{i}\in \left[1,W\right]$ with $P(U_{i})=1/W$. After the random backoff procedure, the channel access is obtained by the node of the *n* competing nodes with the lowest $U_{i}$ value. If we consider station 1 as reference, and without loss of generality, its channel access probability P(win) can be calculated as follows:

$$P\left(win\right)=P(\left(U_{1}<U_{2}\right)\wedge \left(U_{1}<U_{3}\right)\wedge \ldots \wedge \left(U_{1}<U_{n}\right))$$

The above equation is difficult to calculate because the involved variables are dependent on each others. Nevertheless, if an auxiliary random variable *d* is defined as:

$$d=\min\left\{U_{2},U_{3},\ldots ,U_{n}\right\}\in \left[1,W\right]$$

then, the probability that station 1 wins the backoff contention is simply:

(1) $$P\left(win\right)=P\left(U_{1}<d\right)$$

Random variable *d* models the behavior of the rest of stations. Using it, the probability of winning conditioned to *d* can be easily calculated:

$$\begin{array}{cc} P(win|d) & d\\ 0 & 1\\ 1/W & 2\\ 2/W & 3\\ 3/W & 4\\ . & .\\ . & .\\ (W-1)/W & W \end{array}$$

and applying the total probability theorem:

(2) $$\begin{array}{c} P(win)=\sum P(win|d)P(d)=\\ =\frac{1}{W}\sum_{i=2}^{W}\left(i-1)P(d=i\right)=\frac{E[d]-1}{W} \end{array}$$

Now, we obtain the probability of a reference station of winning the channel access when its $CW_{min}$ value is decremented in *k* units, that is, it obtains some advantage with respect to the others (a similar development can be applied in the case that $CW_{min}$ value is increased in *k* units).

If station 1 decreases its $CW_{min}$ in *k* units, then:

$$\begin{array}{c} U_{1}\in \left[1,W-k\right]\\ U_{i}\in \left[1,W\right]\;i\geq 2\\ d=\min\left\{U_{2},U_{3},\ldots ,U_{n}\right\}\in \left[1,W\right] \end{array}$$

with $P(U_{i})=1/W\;\forall i\geq 2$, $P\left(U_{1}\right)=1/\left(W-k\right)$. As before, the probability of winning conditioned to *d* can be calculated:

$$\begin{array}{cc} P(win|d) & d\\ 0 & 1\\ 1/(W-k) & 2\\ 2/(W-k) & 3\\ 3/(W-k) & 4\\ . & .\\ . & .\\ \left(W-k-1)/(W-k\right) & W-k\\ 1 & W-k+1\\ . & .\\ . & .\\ 1 & W \end{array}$$

Therefore,

(3) $$\begin{array}{c} P(win)=\sum P(win|d)P(d)=\\ =\frac{1}{\left(W-k\right)}\sum_{i=2}^{W-k}\left(i-1\right)P\left(d=i\right)+\sum_{i=W-k+1}^{W}P(d=i) \end{array}$$

Finally, the gain *G* associated with the reference station is obtained:

$$G=\frac{{P\left(win\right)|}_{CW_{min1}=default\_CW_{min}-k}}{{P\left(win\right)|}_{CW_{min1}=default\_CW_{min}}}$$

Surprisingly, the calculation of *G* is relatively straightforward. Both terms have been already calculated when the window size is decremented (Equations (2) and (3) respectively). Therefore,

$$\begin{array}{c} G=\frac{\frac{1}{W-k}\sum_{i=2}^{W-k}\left(i-1)P(d=i\right)+\sum_{i=W-k+1}^{W}P(d=i)}{\frac{1}{W}\sum_{i=2}^{W}\left(i-1)P(d=i\right)}=\\ \frac{\frac{1}{W-k}\sum_{i=2}^{W}\left(i-1)P(d=i\right)+\sum_{i=W-k+1}^{W}P\left(d=i\right)\left(1-\frac{i-1}{W-k}\right)}{\frac{1}{W}\sum_{i=2}^{W}\left(i-1)P(d=i\right)}=\\ \frac{W}{W-k}\left[1+\frac{\sum_{i=W-k+1}^{W}\left[(W-k)-(i-1)\right]P\left(d=i\right)}{\sum_{i=2}^{W}\left(i-1)P(d=i\right)}\right] \end{array}$$

and naming the content of the parenthesis 1+ΔG,

(4) $$G=\frac{W}{W-k}\left[1+\Delta G\right]$$

The term ΔG can be expressed as:

$$-\frac{\sum_{i=1}^{k-1}iP(d=W-k+1+i)}{E[d]-1}$$

**Figure 1:**
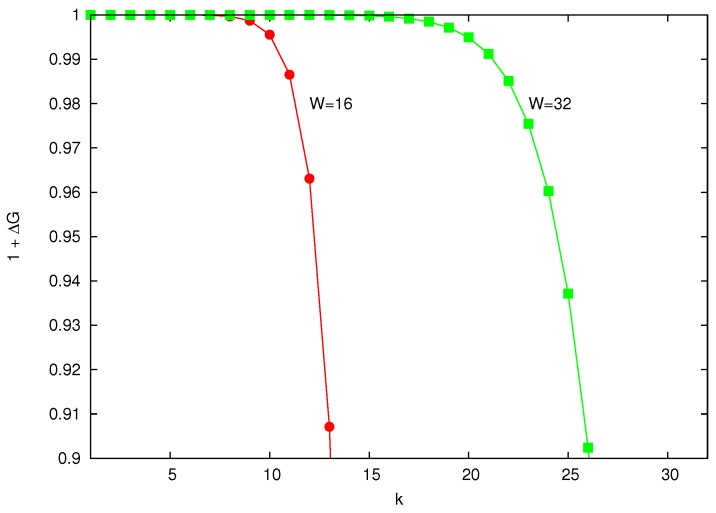
Values of gain 1+ΔG for W={16,32}, that is, $default\_CW_{min}=\left\{15,31\right\}$.

Figure 1 shows the term 1+ΔG as a function of *k* for W={16,32} (that is, $default\_CW_{min}=\left\{15,31\right\}$). It can be seen that the value of 1+ΔG is practically one, even for values of *k* close to *W*. The numerator of ΔG is a weighted average of the last terms of P(d). However, as it is explained in Appendix A, the random variable *d* concentrates its probabilities in the first values of *d*. On the other hand, the numerator is always greater than 1. Thus, ΔG is a value very close to 0 and can be disregarded in Equation (4), and consequently,

(5) $$G=\frac{W}{W-k}=\frac{default\_CW_{min}+1}{default\_CW_{min}+1-k}$$

It can be seen that the expression of *G* does not depend on the random variable *d*, which means that the gain of the reference station does not depend on the rest of stations.

As said before, a similar analysis can be carried out in the case that the $default\_CW_{min}$ value is increased in *k* units, obtaining the following expression:

(6) $$G=\frac{W}{W+k}=\frac{default\_CW_{min}+1}{default\_CW_{min}+1+k}$$

Again, *G* does not depend of the random variable *d*.

Due to the fact that *G* does not depend of the number of competing stations, *G* can be used to propose new solutions that manage QoS requirements in wireless networks. The gain gives us: (1) a measure of the cost/benefit of modifying the default $CW_{min}$, (2) a simple way to measure the competitive advantage gained by a station in front of others, and (3) a scalable strategy to fairly compensate the positive and negative gains around the network.

**Figure 2:**
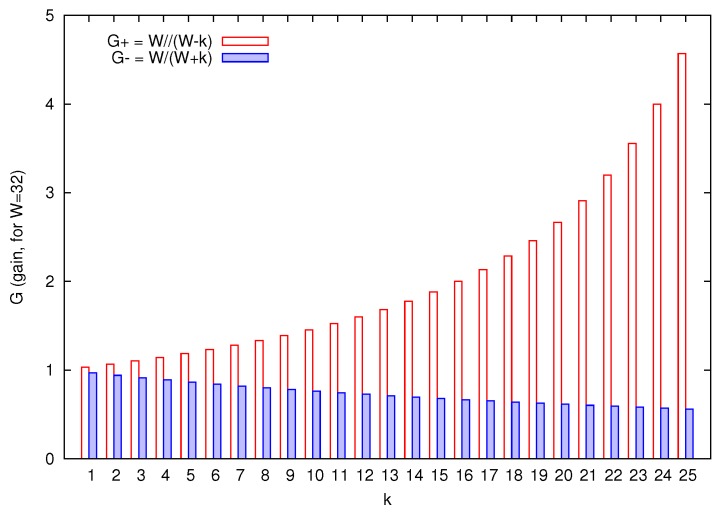
Values of gain *G* when the default value of $CW_{min}$ is decremented in *k* units ($G^{+}$) and incremented in *k* units ($G^{-}$), for W=16.

Figure 2 shows the evolution of the gain *G* when the $CW_{min}$ value is decremented in *k* units (it is represented as $G^{+}$) and when it is incremented in *k* units (it is represented as $G^{-}$). As it can be clearly seen, $G^{+}$ has a quasi-exponential behavior, while $G^{-}$ decreases slowly in a liner way.

The different behavior of $G^{+}$ and $G^{-}$ is going to determine the number of terminals that are required to manage a certain bandwidth redistribution between *requesting terminals* and *giving terminals*. To compensate the gain obtained by a terminal that reduces its contention window size, it will be necessary to increase drastically the contention window size of a *giving* terminal (if it is possible), or even better, to select a group of *giving* terminals for distributing the total increase among them.

Each scenario of application of the proposed DEDCA mechanism will determine design constraints that, known the behavior of $G^{+}$ and $G^{-}$, will allow to obtain the number of involved terminals and the adequate *k* values.

Finally, it is important to point out that the only requirement of the model is the fact that each contending station selects uniformly the duration of its backoff period. No other assumptions are needed. The simplicity of a mathematical model is often the guarantee that it will be valuable in real situations, especially in VANET, WSN and IoT networks.

#### 2.2.2. Gain Calculation in Asymmetric Scenarios

The gain value deduced in Section 2.2.1 supposes that all the stations use the same initial $CW_{min}$ value. However, as a consequence of our QoS-aware medium access control algorithm, it would be possible to find an scenario where nodes have different initial values of $CW_{min}$. In Appendix B, it is developed a mathematical study to show that the gain *G* is practically independent of the initial window size ($CW_{min}$) of the participants (see Equations (A8) and (A9)).

## 3. Proposed SDN-Based Framework for Implementing DEDCA

This section describes a SDN framework for IEEE 802.11 infrastructure-based networks that implements the proposed DEDCA mechanism. Being the central point of the network management and network monitoring, the SDN controller is the ideal network element to manage the processes related to the EDCA mechanism, by programming the adequate software module. This framework makes possible a dynamic management of the backoff values related to contention-based medium access mechanism of the standard IEEE 802.11, offering to the wireless terminals a response to dynamic QoS requirements.

**Figure 3:**
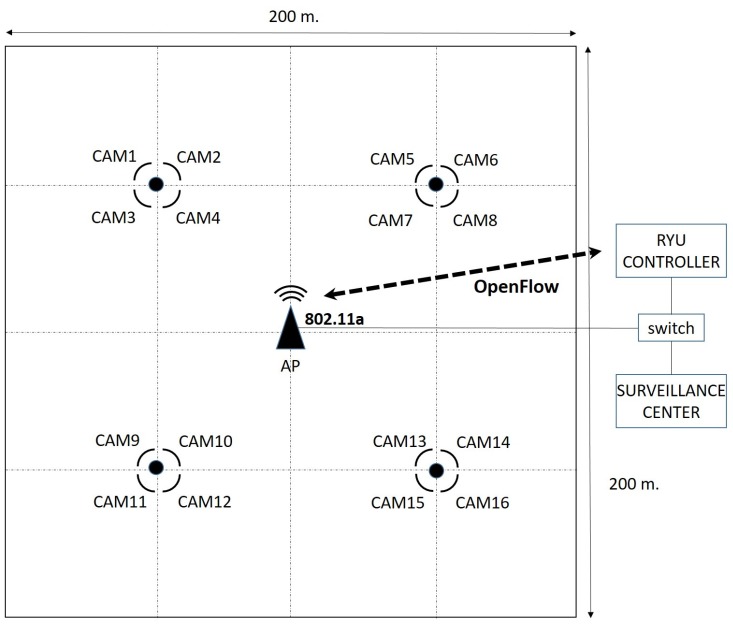
Schematic representation of the outdoor parking lot.

The amount of real scenarios and applications which could benefit from the implementation of the proposed DEDCA mechanism is wide and varied. By way of example, we are going to consider a particular scenario that allows us to show the validity and usefulness of our proposal. In this case, we are going to consider an outdoor parking lot, where a surveillance system is installed. It is represented in a schematic way in Figure 3.

The wireless network consists of an SDN 802.11a Access Point that is equipped with OpenFlow protocol (i.e., OpenWRT AP) and sixteen 802.11a outdoor surveillance cameras that cover a parking lot of dimension 200 m × 200 m. The video signals captured by the cameras are transmitted to a surveillance center, where a surveillance software and the SDN controller are executed.

When all the wireless terminals are configured by default, the available bandwidth is equally shared among all the cameras. The 802.11a data rate is set to 24 Mbps. Thus, due to the fact that there are sixteen contending stations, each station only obtains enough bandwidth to transmit H.264 video streaming at the common frame resolution 4CIF and 12 fps (the typical frame rate in parking lots). If a higher resolution is required by any of the cameras at a given time, the bandwidth sharing has to be modified.

We propose to use the DEDCA mechanism to give a solution to this problem. As detailed in Section 2.2, the DEDCA mechanism is based on the definition of three terminal categories (TC): *normal*, *requesting* and *giving terminals*. In this case, the default backoff configuration of a terminal, which corresponds to a $CW_{min}$ of 31, defines a *normal terminal*. On the other hand, the cameras that require a higher bandwidth are the *requesting terminals* and, finally, the set of cameras that give part of their bandwidth up are the *giving terminals*.

The SDN controller will be in charge of detecting the *requesting terminals* and selecting the set of *giving terminals*. In addition, using the *gain* scalar *G* from Section 2.2.1, the controller will obtain the new backoff values of all these terminals.

### 3.1. Detecting Requesting Terminals

In our scenario, we considered two situations in which a normal terminal can become a *requesting terminal*. The first one considers that there is a sensor-based application associated with each camera that launches an Alarm when something unusual happens. In the second case, it is the security officer at the security center who detects a problematic situation and launches an Alarm. In both cases, the alarm messages, whose structure is *ALARM:0*, are encapsulated into UDP packets. The associated camera will improve its frame resolution. Consequently, a higher bandwidth is needed in order to be able to transmit a video of higher quality.

In a SDN network, the well-known OpenFlow protocol [5] offers a direct communication between the controller and the network elements that allows the programming of the forwarding plane of the network elements. However, terminals and the controller are not directly accessible. In this SDN-based proposed framework, we will use the fully programmable forwarding plane of the SDN application point to solve this limitation, as it is described next.

**Figure 4:**
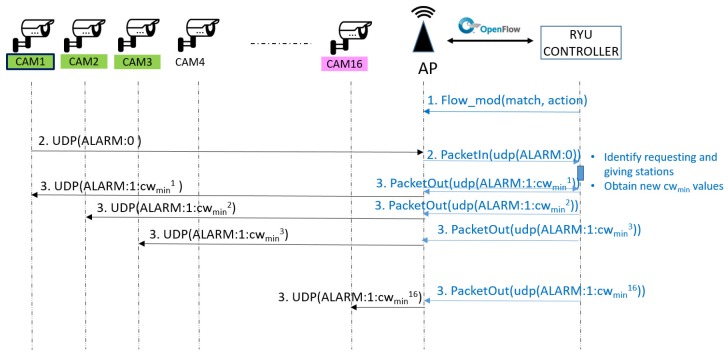
Communication flow of the implemented solution.

The Alarm messages, which are received by the SDN 802.11 Access Point, have to reach the SDN controller, where the system intelligence is located. To enable this to be done, an adequate flow table entry is proactively inserted in the OpenFlow flow table of the access point (step 1 in Figure 4). Thus, the Alarm messages will match the corresponding entry of the flow table at the access point, and will be sent to the controller as *packet_in* messages (step 2 in Figure 4).

The SDN controller, which has a global knowledge of the network (among other parameters, the location of the cameras and their networking configuration), will be able to identify the *requesting station* after receiving the *packet_in* message and parsing the encapsulated packet.

### 3.2. Selecting Giving Terminals

As said before, the aim objective of our solution is to change the way the available bandwidth is shared among the cameras in order to allow a *requesting camera* to transmit the video with a higher resolution. In this scenario we want to improve the resolution from 4CIF@12fps to 720p@12fps, which means to double the required data rate.

If the default value of $CW_{min}$ is set to 31 in all the cameras, from Equation (5), it can be deduced that the value of $CW_{min}$ of the *requesting camera* should be reduced in 16 units in order to double the probability of winning the channel.

Because the *requesting camera* improves its bandwidth, the rest of cameras will get worse. However, as it is studied by simulation in Section 4.1.1, if a subset of cameras are chosen to compensate the increase in the gain of the *requesting terminals*, the remaining nodes will not notice any change in their channel access rate. It does not matter how the compensation is done as long as the increment in the obtained bandwidth of the *requesting terminal* is equal to the decrement in the obtained bandwidth of the *giving terminals* (see Equation (7)).

**Figure 5:**
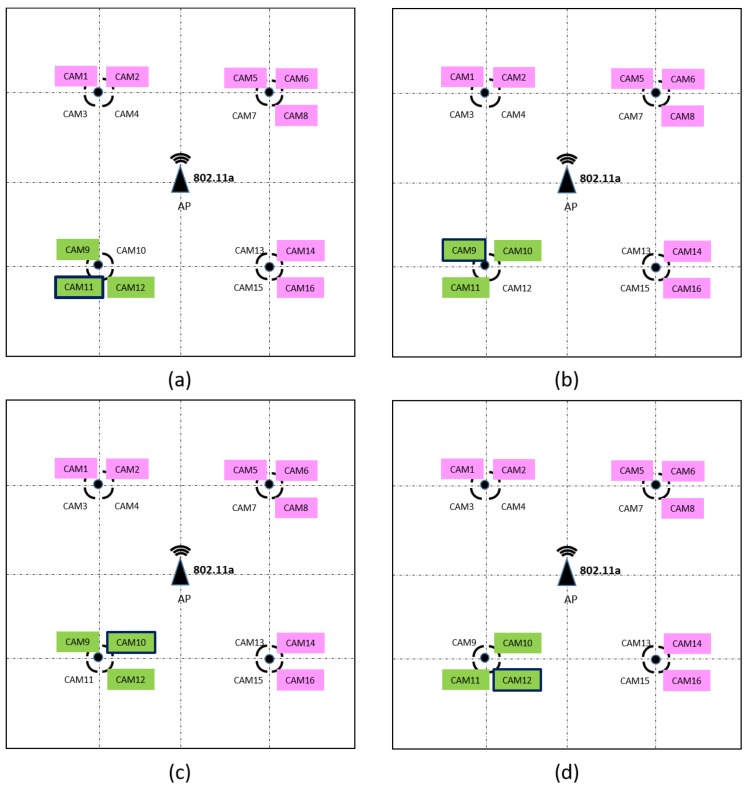
The choice of *requesting* and *giving stations* depending on the camera that generates the ALARM. Due to the symmetry of the scenario only 4 cases are shown. (**a**) CAM11 generates the ALARM. (**b**) CAM9 generates the ALARM. (**c**) CAM10 generates the ALARM. (**d**) CAM12 generates the ALARM. The cameras marked in green are the *requesting stations* and the cameras marked in pink are the *giving stations*.

This behavior has been taking into account to define the algorithm that the controller applies to select the *giving terminals* in this scenario and to obtain their required $CW_{min}$ values. When a camera launches an Alarm, the SDN controller will consider as *requesting terminals* this camera and its two neighbor cameras. This will enable to increase the video resolution of these cameras, offering to the security officer a more detailed view of the conflict area. In addition, the controller hast to select the *giving stations* and to obtain their new $CW_{min}$ values. In this implementation, we have chosen that the *giving stations* are the seven furthest cameras, as it is shown in Figure 5. Finally, applying Equation (7), the new $CW_{min}$ value of these terminals is obtained.

In this case, the number of stations s=16, and the number of *requesting stations* and *giving stations* are set to r=3 and g=7, respectively. As the objective of the requesting stations is to duplicate the obtained bandwidth, $G^{+}=2$. Consequently, applying Equation (7) it can be obtained that the *giving stations* have to increase their $CW_{min}$ value in k=24 units, so their $CW_{min}$ value will be increased to 55.

### 3.3. How to Communicate the Configuration Changes

Once *requesting* and *giving terminals* have been identified and the new $CW_{min}$ values have been obtained by the SDN controller, it is necessary to communicate the new configuration to the cameras.

As said before, there is no a direct communication channel between the controller and the stations. Again, we are going to use OpenFlow protocol and the programmable capability of the SDN access point to solve this limitation.

For each *requesting* and *giving terminal*, the controller will create an ALARM response message, whose structure is $ALARM:1:cw_{min}\_value$. As in the case of the Alarm messages, the Alarm response message is encapsulated into an UDP packet that is sent to the corresponding station by the Access Point. To force the sending, the SDN controller will generate *packet_out* messages that contain the frame in the payload field, and indicate the instruction of sending the frame to the wireless port (step 3 in Figure 4).

The stations, that are listening for UDP packets on the predefined port, will receive the Alarm response and will apply the new $CW_{min}$ configuration by means of modifying its value in the network-device firmware (see Section 4.2.1).

This procedure will be used also to restore the initial configuration of the *requesting* and *giving terminals* once the high-demanding situation, generally of limited duration, has finished.

## 4. Evaluation

To provide a detailed and clear evaluation of the overall system, this section has been divided into two parts. The first one is focused on the evaluation of the most important aspects of the mathematical model. The second part is used to evaluate the proposed SDN-based framework.

### 4.1. Evaluation of the Mathematical Model

#### 4.1.1. Impact of DEDCA mechanism in the *normal terminals*

Equations (5) and (6) describe the multiplicative increment and decrement in the probability of winning the channel, when the $CW_{min}$ value of the *requesting* and *giving terminals* are modified. However, it would be interesting to know how the *normal* stations are affected.

In a backoff-based network, the bandwidth is shared among all the stations. Therefore, assuming that the network has a capacity of C bps, the bandwidth assigned to each station is:

C×P(win)

For a *normal* station, the probability of winning the channel has been obtained in Equation (2). However, considering an ideal arbitration of the multiple access control, all the stations tend to obtain the same access probability, and thus this probability can be approximated to:

$$P{\left(win\right)}_{normal}\approx \frac{1}{s}$$

where *s* is the number of stations.

On the other hand, the probabilities that a *requesting station* and a *giving station* win the channel are respectively:

$$P{\left(win\right)}_{requesting}=P{\left(win\right)}_{normal}\cdot G^{+}=\frac{1}{s}\cdot \frac{default\_CW_{min}+1}{default\_CW_{min}+1-k}$$

$$P{\left(win\right)}_{giving}=P{\left(win\right)}_{normal}\cdot G^{-}=\frac{1}{S}\cdot \frac{default\_CW_{min}+1}{default\_CW_{min}+1+k}$$

Adding all the bandwidth assigned to each station:

$$C=\sum_{z=1}^{n}C\cdot \frac{1}{S}+\sum_{i=1}^{r}C\cdot \frac{1}{S}\cdot \frac{default\_CW_{min}+1}{default\_CW_{min}+1-k_{i}}+\sum_{j=1}^{g}C\cdot \frac{1}{S}\cdot \frac{default\_CW_{min}+1}{default\_CW_{min}+1+k_{j}}$$

$$C=n\cdot \frac{C}{S}+\frac{C}{S}\sum_{i=1}^{r}\frac{default\_CW_{min}+1}{default\_CW_{min}+1-k_{i}}+\frac{C}{S}\sum_{j=1}^{g}\frac{default\_CW_{min}+1}{default\_CW_{min}+1+k_{j}}$$

and finally, dividing by C:

(7) $$1=\frac{n}{S}+\frac{1}{S}\sum_{i=1}^{r}\frac{default\_CW_{min}+1}{default\_CW_{min}+1-k_{i}}+\frac{1}{S}\sum_{j=1}^{g}\frac{default\_CW_{min}+1}{default\_CW_{min}+1+k_{j}}$$

where *s* is the number of stations, *r* is the number of *requesting stations*, *g* is the number of *giving stations*, and n=s−r−g is the number of *normal stations*.

In the proposed system, Equation (7) can be used to obtain the number of *giving terminals* and their parameters, given a certain number of *requesting terminals* and the corresponding requested bandwidth increase.

Now, we are going to evaluate the impact of DEDCA mechanism in the normal terminals by simulation. We consider a set of s=15 terminals whose default $CW_{min}$ is set to 31.

To test DEDCA impact, we have performed multiple simulations as described. For each simulation, a random number of *requesting terminals* are chosen, whose $CW_{min}$ value is decremented in *k* units. Taking into account that the bandwidth increase of *requesting terminals* is going to be compensated by the *giving* terminals, the number of *giving terminals* and their parameters are obtained from Equation (7).

The channel access rate of each terminal is obtained considering saturated conditions. All the terminals always have a packet to be transmitted, and they contend for the channel accordingly to their congestion window size.

**Figure 6:**
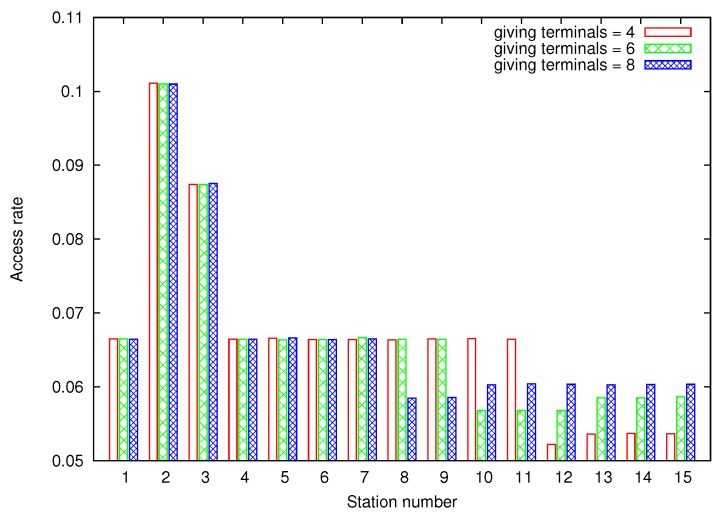
Obtained access rate under saturated conditions. Stations 2 and 3 are fixed as *requesting stations*. 4, 6 and 8 terminals are chosen as *giving terminals* to compensate the gain.

To simplify the drawing of the obtained results, but without loss of generality, Figure 6 shows the simulation results in a particular case, where station 2 and 3 are chosen as *requesting stations*. The default $CW_{min}$ value of these *requesting stations* is decremented in 10 and 7 units (that is, the new $CW_{min}$ value is set to 21 and 24), respectively. The figure plots the access rate obtained by each one of the 15 terminals when the gain of the *requesting stations* is compensated in three different ways, selecting five, six and eight *giving terminals* respectively. From Equation (7), the default $CW_{min}$ values of the *giving stations* have to be incremented in 8,7,7,7 units in the first case; in 5,5,5,4,4,4 in the second case, and in 4,4,3,3,3,3,3,3 in the last case. As can be seen, the fact that *giving* and *requesting terminals* modify their backoff values, and the way this modification is done, does not affect the channel access rate of the *normal* terminals.

#### 4.1.2. Tail latency

As said before, the IEEE 802.11 network family has become a common wireless solution for data networks. However, most of the services running on top of these networks (particularly VANET, WSN and IoT) are delay sensitive applications, being very sensitive to tail latency.

Tail latency, defined as the delay between consecutive frames, has been studied recently by [6]. Because the proposed DEDCA mechanism is focused on providing response to dynamic QoS requirements, even in delay-sensitive environments, it is important to test the behavior of this parameter when DEDCA is applied.

**Figure 7:**
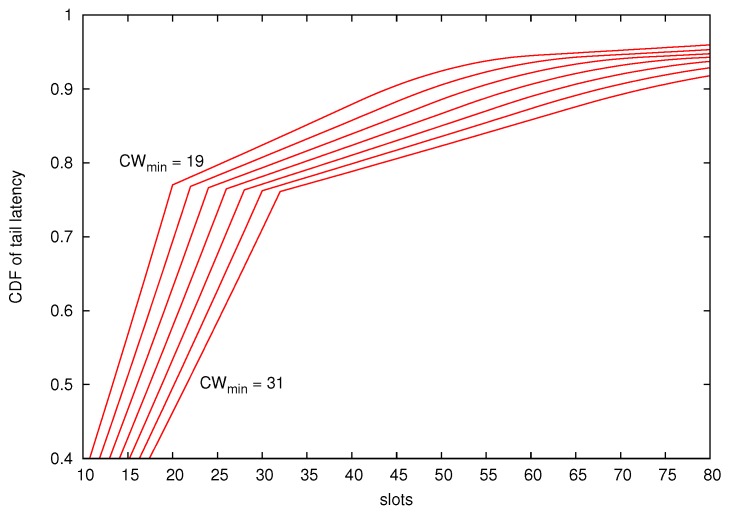
CDF of the Tail latency. The number of competing stations is set to n=10.

Here, we consider 10 stations competing for the channel. The default $CW_{min}$ is set to 31 for all of them. However, in each simulation, station one decrements its $CW_{min}$ value by two units from 29 to 19 (concretely $CW_{min}=\left\{29,27,25,23,21,19\right\}$). Figure 7 shows the *Cumulative Distribution Function* (CDF) of the tail latency at station one. As can be seen, the use of the proposed DEDCA mechanism enables to achieve a noticeable reduction of the tail latency. For example, the probability that the tail latency is below 25 slots is around 0.8 when $CW_{min}=19$, but only above 0.6 when $CW_{min}=31$.

Although the solution proposed in [6] is a solution specifically designed to be used in scenarios where real-time services are dominant, the proposed mechanism DEDCA is able to reach similar performances and, as it has been pointed out, it can be used -at least temporarily- in a wireless network with a mixture of services in terms of QoS.

### 4.2. SDN-Based Framework Implementation

#### 4.2.1. Implementation Issues

The IEEE 802.11 backoff standard forces that any value of the $CW_{min}$ must be always a power of 2 less 1 (i.e., 31, 63, 127, 255, 511, and 1023). Therefore, the $CW_{min}$ value is an all 1’s binary number of length *m*. In this way, the algorithm to generate a random number in this range is simple and easily implementable.

It is clear if our proposed DEDCA mechanism does not satisfy this requirement. However, this is not a big obstacle, neither when using simulations nor in Software Defined Radio (SRD) [7], since it is quite simple to modify the software code.

On the other hand, most hardware manufacturers develop the backoff protocol in a binary microcode firmware or in proprietary kernel modules, which are loaded by the operating system directly into device memory. Although this complicates significantly any change of the default protocol behavior, it is still possible to use an unrestricted value for $CW_{min}$, as authors of [8] demonstrate using Broadcom B43 wireless card. The changes in the assembly language of the backoff algorithm can be easily exported by manufacturers to any other wireless card.

We have implemented the proposed DEDCA mechanism in a testbed consisting of a Ryu SDN controller and an 802.11 Access Point equipped with OpenFlow v. 1.3 using the simulator Estinet 9.0 OpenFlow Network Simulator [9].

#### 4.2.2. Simulation Results

Unlike other tools used in the field of SDN research, such as the well-known Mininet emulator, Estinet 9.0 can be used to evaluate accurately a wide set of properties of the links that connect the OpenFlow network elements. In this work, Estinet 9.0 SDN Wifi-Infrastructure simulation mode offered us the requited control over the IEEE 802.11a protocol implementation.

Considering the parking slot scenario, multiple situations could be taken into account according to the camera that launches the Alarm. By way of example, this section shows the response of the implemented system when CAM11 generates the Alarm message. The 802.11a data rate is set to 24 Mbps, and the initial value of $CW_{min}$ is set to 31 (the protocol default value).

**Figure 8:**
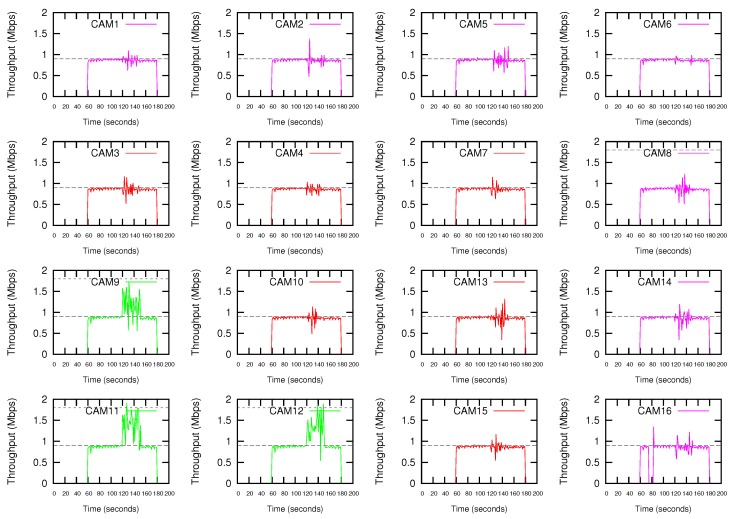
Throughput without using the proposed DEDCA mechanism. CAM11, CAM9 and CAM12 switch to high-demanding transmission mode after CAM11 launches an ALARM.

Due to the missing of Wifi cameras in the simulator tool, we have used the *Iperf* tool to test the bandwidth performance of the cameras. In the normal transmission mode, each terminal generates an UDP stream at a rate of 0.9 Mbps. The data rate required by the transmission of 4CIF@12 video streaming is around this value. A 720p@12fps video streaming requires twice the data rate. In the high-demanding transmission mode, each terminal generates an UDP stream at a rate of 1.8 Mbps. Figure 8 shows the throughput obtained by the Wifi cameras when the proposed DEDCA mechanism is not applied. All the cameras start the transmission, in the normal transmission mode, at second 60. At second 120, CAM11 launches an ALARM, and as a result, CAM11, CAM9 and CAM12 switch to the high-demanding transmission mode that will last for 30 s. It can be seen that these three cameras increase their bandwidth, but the required value is not reached. This increment is due to the fact that, considering the 802.11a data rate of 24 Mbps, there is a remaining bandwidth, but it is not enough to respond to the demand of the requesting stations.

**Figure 9:**
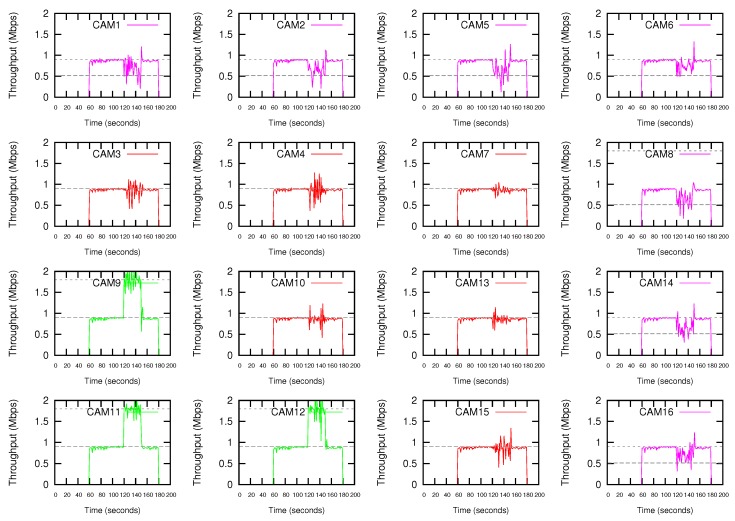
Throughput using the proposed DEDCA mechanism. CAM11, CAM9 and CAM12 switch to high-demanding transmission mode after CAM11 launches an ALARM.

Figure 9 shows the results obtained if the proposed DEDCA mechanism is applied. Once the ALARM is sent by CAM11, the SDN-based infrastructure allows the SDN controller to realize that a high-demanding transmission is required. The adequate software module determines the *requesting cameras*, and making use of the scalar G defined in the proposed DEDCA mechanism, is also able to select the *giving cameras* and to obtain required modifications of the contention window size of these stations. Again, the SDN-based infrastructure allows the controller to inform the stations about their new configuration values.

After modifying their $CW_{min}$ value adequately, the requesting cameras (CAM9, CAM11 and CAM12) are able to reach the demanding bandwidth. On the contrary, as it was expected, the seven furthest cameras (CAM1, CAM2, CAM5, CAM6, CAM8, CAM14 and CAM16) suffer a decrement of the throughput due to the increment of their $CW_{min}$ value. The remaining six cameras (CAM3, CAM4, CAM7, CAM10, CAM13 and CAM15) are considered *normal terminals*. Consequently, due to the fact that their $CW_{min}$ value is not modified, their performance is maintained.

It can be also observed that the *giving* cameras actually reach a throughput slightly higher than the expected. The reason is the remaining bandwidth that we could observed in Figure 8. The complete data rate (i.e., 24 Mbps) was not consumed by stations. Now, thanks to the application of the DEDCA mechanism, the *requesting cameras* obtain the required bandwidth. The *normal cameras* are not affected and still obtain the adequate throughput. Finally, the *giving terminals* reduce their throughput, but CSMA/CA distributes the remaining rate among them.

Finally, it can be observed that, as usual in shared networks, the throughput oscillates. However, during the first period of time (from 60 to 120 s) it is rather stationary, mainly due to the fact that it this period there is enough bandwidth in the network for all competing stations.

## 5. Related Works

Much of the research in wireless SDN has focused in IEEE 802.11 networks. An important feature of SDN-enabled WLANs is virtualization. The ability to slice the network, based on users, subnets or traffic, allows many benefits [10]. Another research interest in this area is related to mobility management. For example, Odin [11] is an SDN framework that proposes to simplify the implementation of authentication, authorization and accounting (AAA), by moving to a centralized architecture, which eases to implement mobility managers and to mitigate the hidden terminal problems. On the other hand, there have been multiple research efforts in multi-hop networks. Ref. [12] proposed an architecture for wireless mesh networks with traffic management functionality. In this architecture, the control plane is composed by four modules: global overview manager, routing path computation, traffic scheduling, and lastly spectrum allocation to configure radio resources. In addition to this, routers are also equipped with a monitor module to send neighbor connectivity information to the global overview manager at the controller and a radio frequency tuning module for assigning the radio frequency to transmit the data and control traffic.

Regarding QoS management in SDN networks, the rule placement and caching in SDN switches have been studied in [13,14]. Given a set of sessions with certain QoS requirements, instead of installing all the forwarding rules and QoS requirement rules in every switch, the authors of [13] divide a session into several independent sub-sessions with different subsets of QoS requirement rules, which are scattered across multiple switches, to reduce the TCAM usage. In [14], the authors studied the rule caching problem in SDN in order to minimize the amount of TCAM and remote controller processing cost. Finally, in [15] authors studied a problem in which the load balancing of the control plane traffic and the cost of stablishing the control channel are jointly considered when selecting the protection paths for control channels. Simulation results show that the algorithm has high efficiency in resource utilization.

The main contribution of this work refers to the media access control mechanism of 802.11 networks. An analytical model of the IEEE 802.11 CSMA/CA mechanism was presented in [16,17]. Based on a complex bi-dimensional Markov Chain, this model has been widely used in the literature as a theoretical corpus to calculate different set of parameters related to CSMA/CA performance evaluation. The model has also been extended to include priorities [18], high traffic conditions [19] and loss channels [20].

With regard to the modification of the $CW_{min}$ value, different solutions have been proposed. These works differ in the parameters that are used to calculate the new value, where the modification is calculated (in the access point or the stations) or when the $CW_{min}$ value is updated.

In [21], the $CW_{min}$ value of each Access Category defined in IEEE 802.11e is adapted according to the traffic load and channel conditions. A higher value is set when the channel is estimated to be congested and a smaller value is set when the channel load is estimated to be low. The $CW_{min}$ value is obtained at each station (that is, in a distributed way), considering the instantaneous collision rate (calculated using the number of collisions and the number of packets sent during a period of time) experimented during the update period. Therefore, there does not exist any coordination between the stations that increase and decrease their $CW_{min}$ values. On the contrary, in our proposal, the mechanism that determines how to modify the $CW_{min}$ value of the stations is implemented in a centralized way.

Ref. [22] proposes a $CW_{min}$ adaptation mechanism which takes place periodically at the access point. In this case, all the stations update the $CW_{min}$ parameter to the same value, which is computed at the access point. This $CW_{min}$ parameter is computed considering the maximum collision probability among all the stations, which report an explicit feedback to the access point containing measurements of their individual collision probabilities. If the maximum collision probability exceeds an upper control threshold, the $CW_{min}$ is doubled respect to the behavior defined by the IEEE 802.11e standard. If the probability is below a lower threshold, the $CW_{min}$ is decreased by half. After a contention window adaptation, the algorithms waits for a period τ before changing the $CW_{min}$ value again. In this proposal, the network resources are not properly used. Assuming that all the stations have the same behavior is not a good statement, considering that in a wireless medium the channel conditions can only change for some stations.

In general, the $CW_{min}$ adaptation mechanisms that are executed periodically can be divided into two types: *Multiplicative Increase-Multiplicative Decrease* (MIMD) and *Additive Increase-Additive Decrease* (AIAD) schemes. Authors in [23,24,25,26] propose MIMD solutions. The multiplication factor is defined by a function of the priority and the collision rate, or simply by using a fixed value. Authors in [21,27,28] use AIAD schemes. The additive changes of the contention window depend on the collision rate, the priority, or simply fix values are used.

On the other hand, the modification of the $CW_{min}$ parameter has been applied in different environments. [29,30] try to solve the throughput unfairness problem inherent to WiFi networks where devices of *a/b/g* and *n* types can coexist. Slow stations occupy the channel more time to transfer the same amount of data. This fact degrades significantly the performance of high-speed stations and decreases the overall network throughput. To solve this problem, faster stations should get a higher chance of accessing the medium (i.e., get more transmission opportunities) than slower ones. This can be achieved via scaling down their contention windows or by scaling up contention windows used by slow stations. However, this proposal only takes into account the WiFi standard of every station, that is, it does not take into account the channel conditions at all.

In multihop wireless networks, packets from a source node are relayed by intermediate nodes (relay nodes) towards their destination along a multihop wireless path. Ideally, in this scenario, a node should not transmit to the relay node more packets than the relay node can forward. In [31], authors propose a fully distributed contention window adaptation mechanism that adjusts the channel access probability depending on the difference between the incoming and outgoing traffic at each node, in order to equate the traffic forwarding capabilities among all the nodes in the path.

Ref. [32] considers vehicular networks based on IEEE 802.11. In this scenario, it is proposed to adjust the $CW_{min}$ parameter depending on the local node density. For that, five different mechanisms for local density estimation are proposed and evaluated. Results show the efficiency and simplicity of such mechanisms, so they recommended as building blocks of any architecture for VANET congestion control.

There are a set of works that propose to obtain the exact value of the CW in the backoff procedure instead of just modify the $CW_{min}$ parameter.

In [33], authors propose to turn off the binary exponential backoff algorithm of IEEE 802.11 and use an appropriate fixed value for *W*, according to the node density and the instantaneous PER. To estimate the number of competing stations (*M*), stations should measure two variables: the number of slots in which the station does not transmit, and the number of busy slots whose energy level measured is higher than a predefined value. Then, a central controller tunes the value of CWratio (the ratio between CW and *M*) periodically, searching for the optimal CWratio. This value is sent to the network and then each station sets its contention window size according to its *M* value, i.e., CW=M×CWratio.

In [34] authors analytically derive the CW value that maximizes the throughput under both saturated and non-saturated conditions. Then, they propose a distributed algorithm to be executed each time there is a packet on the buffer of a station to be transmitted or when a collision occurs.

Refs. [35,36,37] propose mechanisms to tune the CW by observing the channel status. Ref. [38] proposes to dynamically adjust the CW size considering channel bit error rate (BER). However, these methods have to estimate the number of active stations.

Finally, ref. [6] proposes a modification of CSMA/CA contention protocol to mitigate inter-packet latency (also known as tail latency) in IEEE 802.11-based networks. Here, it is guaranteed that every competing node transmits a packet in a round (virtual time slot). The rounds are implemented in a distributed way by the definition of two non-overlapped contending ranges, one of them [0,α) and the other [α,W). As nodes success in their transmissions, they change the contending range from the first range to the second one. However, this solution is still focused on providing QoS in scenarios where real-time services are dominant.

## 6. Conclusions

Nowadays, IEEE 802.11 technology is used in a wide variety of scenarios, such as mobile, vehicular or sensor networks. In all of them, the transmission of real-time and multimedia traffic is very common, and the corresponding applications require that the network supports some minimum QoS requirements. One of the methods to provide it is by giving more channel access opportunities to nodes involved in such sessions. This is the background idea of EDCA, the contention-based medium access mechanism of IEEE 802.11e, that provides long-term Qos.

However, in some situations it is possible that some multimedia traffic sessions require additional QoS guarantees during short-term intervals. For these situations, in this work we have proposed DEDCA. In DEDCA, stations are classified in three different Terminal Categories (TC): *normal stations*, *requesting stations* and *giving stations*. By default, all the terminals are classified as *normal stations*, using default $CW_{min}$ values. Stations that require additional QoS guarantees are called *requesting stations*, and they will modify their $CW_{min}$ values to increment their channel access opportunities, at the expense of *giving stations* that they will modify their $CW_{min}$ value to decrement their channel access opportunities. With the goal of determining how to modify the $CW_{min}$ value of the *giving* and *requesting stations*, a mathematical model has been developed. This model is able to determine the number *requesting stations* and *giving stations* that are required to manage a certain QoS requirement.

The previous processes related to the DEDCA mechanism need to be implemented in a centralized way, in order to be able to identify the giving and requesting stations in the network. Controllers of Software-Defined Networks have a centralized control over the network, and they seem to be the ideal elements to implement this mechanism. In this paper, that is the reason we have proposed a SDN framework for IEEE 802.11 networks that implements the proposed DEDCA mechanism. In this framework, the SDN controller is attached to the IEEE 802.11 access points. The communication between the SDN controller and the access points is based on OpenFlow protocol and the flow tables of access points have an appropriate flow entry in order to forward the UDP alarm messages generated by the requesting stations to the SDN controller. On the other hand, the SDN controller will generated *packet_out* messages with the new $CW_{min}$ values for *giving* and *requesting stations*. These messages will be encapsulated in UDP segments by the access points and they will be forwarded to the corresponding stations.

The proposed SDN framework has been evaluated by simulation using the Estinet network simulator. We have considered an outdoor parking lot, where a surveillance system is installed. The simulations results show that after modifying their $CW_{min}$ value adequately, the requesting cameras are able to reach the demanding bandwidth. On the contrary, as it was expected, the giving cameras suffer a decrement of the throughput due to the increment of their $CW_{min}$ value. The remaining cameras are considered normal stations. Consequently, due to the fact that their CWmin value is not modified, their performance is maintained.

## Figures and Tables

**Figure A1:**
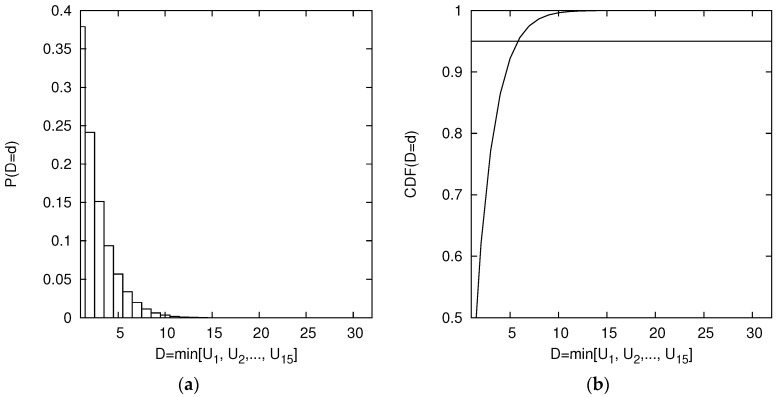
(**a**) Distribution of P(D=d) and (**b**) Cumulative Distribution Function CDF(D=d) for the case $CW_{min}=32$ and number of competing station n=15. The 95% of the probability of *D* is concentrated in d>=6. In addition, the probability is almost 0 from d≈10.

## Appendix A. Mininum Random Variable

Throughout the analysis it will be often used a random variable defined as the minimum of a set of independent random variables. Therefore, it is convenient to sum up the main formulas related to this random variable.

Let be the random variable $D=\min\{U_{1},U_{2},\ldots ,U_{n}\}$, where $U_{i}$ are independent random variables. The density function of *D* is:

F(D)=P(D≤d)=1−P(D>d)

Due to *D* is the minimum of $U_{i}$, all $U_{i}$ must be necessarily greater than *d*,

$$F\left(D\right)=1-P(\left(U_{1}>d\right)\wedge \left(U_{2}>d\right)\wedge \ldots \wedge \left(U_{n}>d\right))$$

Since the random variables $U_{i}$ are independent each other,

$$F\left(D\right)=1-\prod_{i=1}^{n}P\left(U_{i}>d\right)=1-\prod_{i=1}^{n}\left[1-F(U_{i})\right]$$

If $U_{i}$ are i.i.d uniform random variables,

$$P\left(U_{i}\right)=1/CW_{min}\Rightarrow F\left(U_{i}\right)=\frac{d}{CW_{min}}\;1\leq d\leq CW_{min}$$

Then the density and probability functions of D are,

(A1) $$\begin{array}{c} F\left(D\right)=1-{\left[1-\frac{d}{CW_{min}}\right]}^{n} \end{array}$$

(A2) $$\begin{array}{c} P\left(D=d\right)={\left[1-\frac{d-1}{CW_{min}}\right]}^{n}-{\left[1-\frac{d}{CW_{min}}\right]}^{n} \end{array}$$

Here, we want to point out two important aspects of the random variable *D*. First, as it can be foreseen taking into account the definition of the *minimum*, the probabilities are concentrated in the first values of *d*. A simple way the proof this is by finding the value of $d_{th}$ that satisfies:

(A3) $$F(D>d_{th})>x$$

where *x* is usually located in $\left[0.9,1\right)$. From Equations (A1) and (A3):

(A4) $$d_{th}>CW_{min}\left(1-\sqrt[{n}]{\left(1-x\right)}\right)$$

For example, when there are n=15 stations competing for the channel, the 95% of the probability of *D* is concentrated approximately in the first $0.39\cdot CW_{min}$ values of *d*. This means that P(D) follows a exponential distribution, as can be seen in Equation (A2) and Figure A1, that shows P(D) when $CW_{min}=32$.

The second interesting aspect of the *minimum* function is the complexity. Both, the density and the probability functions depend on the number of competing station *n* and the maximum value of the backoff $CW_{min}$. It is not easy to obtain a real-time trace of *n* since although the number of playing stations can be assumed fixed, *n* represents the number of stations competing for the channel at the same time. On the other hand, as it will be shown later, in a QoS scenario each station can have its own value of *W*, increasing even more the complexity of the equations.

## Appendix B. Gain in Asymmetric Scenarios

Here, it is suppose that each station has its own window size, independently of the others. For simplicity, only the case in which the window size is decreased in *k* unit will be developed (the other case can be easily calculated following the same methodology).

$$\begin{array}{c} \forall i,\;U_{i}\in \left[1,W_{i}\right]\;\mathrm{with}\;P\left(U_{i}\right)=1/W_{i}\\ d=\min\left\{U_{2},U_{3},\ldots ,U_{n}\right\}\in \left[1,W_{min}\right] \end{array}$$

where

$$W_{min}=\min\left\{W_{2},W_{3},\ldots ,W_{n}\right\}$$

As usual, without loss of generality, the station 1 is taking as reference. The window size of station 1 could be $W_{1}\geq W_{min}$ or $W_{1}<W_{min}$.

### Appendix B.1. Case I: W_1_ ≥ W_min_

In the first place, it is calculated the probability of winning the channel P(win) under normal circumstances, following the same methodology applied in Section 2.2.1:

$$\begin{array}{cc} P(win|d) & d\\ 0 & 1\\ 1/W_{1} & 2\\ 2/W_{1} & 3\\ 3/W_{1} & 4\\ . & .\\ . & .\\ . & .\\ \left(W_{min}-1\right)/W_{1} & W_{min} \end{array}$$

and applying the total probability theorem:

(A5) $$P\left(win\right)=\frac{1}{W_{1}}\sum_{i=2}^{W_{min}}\left(i-1)P(d=i\right)$$

Now, it is supposed that station 1 has decreased its window size in *k* units. The P(win) can be calculated as:

$$\begin{array}{cc} P(win|d) & d\\ 0 & 1\\ 1/(W_{1}-k) & 2\\ 2/(W_{1}-k) & 3\\ 3/(W_{1}-k) & 4\\ . & .\\ . & .\\ \left(W_{min}-1\right)/\left(W_{1}-k\right) & W_{min} \end{array}$$

Therefore, applying again the total probability theorem:

(A6) $$P\left(win\right)=\frac{1}{\left(W_{1}-k\right)}\sum_{i=2}^{W_{min}}\left(i-1)P(d=i\right)$$

Now, it is possible to calculate the gain *G* as the quotient between Equations (A5) and (A6) :

$$G=\frac{W_{1}}{W_{1}-k}$$

Which coincide with the symmetric case.

### Appendix B.2. Case II: W_1_ < W_min_

As before, it is calculated the probability of winning the channel P(win) under normal circumstances. In this case, however:

$$\begin{array}{cc} P(win|d) & d\\ 0 & 1\\ 1/W_{1} & 2\\ 2/W_{1} & 3\\ . & .\\ . & .\\ . & .\\ \left(W_{1}-1\right)/W_{1} & W_{1}\\ 1 & W_{1}+1\\ 1 & W_{1}+2\\ . & .\\ . & .\\ . & .\\ 1 & W_{min} \end{array}$$

and applying the total probability theorem:

(A7) $$P\left(win\right)=\frac{1}{W_{1}}\sum_{i=2}^{W_{1}}\left(i-1)P(d=i\right)+\sum_{i=W_{1}+1}^{W_{min}}P(d=i)$$

As in the later case, it is supposed that station 1 has decreased its window size in *k* units. Now, the P(win) can be calculated as:

$$\begin{array}{cc} P(win|d) & d\\ 0 & 1\\ 1/(W_{1}-k) & 2\\ 2/(W_{1}-k) & 3\\ . & .\\ . & .\\ . & .\\ \left(W_{1}-k-1\right)/\left(W_{1}-k\right) & W_{1}-k\\ 1 & W_{1}-k+1\\ . & .\\ . & .\\ . & .\\ 1 & W_{min} \end{array}$$

and applying the total probability theorem:

(A8) $$P\left(win\right)=\frac{1}{W_{1}-k}\sum_{i=2}^{W_{1}-k}\left(i-1)P(d=i\right)+\sum_{i=W_{1}-k+1}^{W_{min}}P(d=i)$$

The gain *G* can be calculated dividing Equation (A8) by Equation (A7),

$$\begin{array}{c} G=\frac{\frac{1}{W_{1}-k}\sum_{i=2}^{W_{1}-k}\left(i-1)P(d=i\right)+\sum_{i=W_{1}-k+1}^{W_{min}}P(d=i)}{\frac{1}{W_{1}}\sum_{i=2}^{W_{1}}\left(i-1)P(d=i\right)+\sum_{i=W_{1}+1}^{W_{min}}P(d=i)}=\\ \frac{W_{1}}{W_{1}-k}\left[1+\frac{-\sum_{i=1}^{k-1}iP\left(d=W_{1}-k+1+i\right)-k\sum_{W_{1}+1}^{W_{min}}P(d)}{\sum_{i=2}^{W_{1}}\left(i-1)P(d=i\right)+\sum_{i=W_{1}+1}^{W_{min}}P(d=i)}\right]= \end{array}$$

and calling the content of the parenthesis 1+ΔG,

(A9) $$G=\frac{W_{1}}{W_{1}-k}\left[1+\Delta G\right]$$

The denominator value of ΔG is very close to E[d], which is greater than one. On the other hand, the probabilities of the numerator are far away from the initial values of *d*. As a consequence,

$$G\approx \frac{W_{1}}{W_{1}-k}$$
